# Probe-Based Confocal Laser Endomicroscopy for Indeterminate Biliary Strictures: Refinement of the Image Interpretation Classification

**DOI:** 10.1155/2015/675210

**Published:** 2015-03-12

**Authors:** Michel Kahaleh, Marc Giovannini, Priya Jamidar, S. Ian Gan, Paola Cesaro, Fabrice Caillol, Bernard Filoche, Kunal Karia, Ioana Smith, Monica Gaidhane, Adam Slivka

**Affiliations:** ^1^Gastroenterology & Hepatology, Weill Cornell Medical College, New York, NY 10021, USA; ^2^Endoscopic Unit, Paoli-Calmettes Institute, 232 Boulevard de Sainte Marguerite, 13273 Marseille Cedex 9, France; ^3^Division of Digestive Diseases, Yale University, New Haven, CT 06520, USA; ^4^Department of Gastroenterology, Digestive Disease Institute, Virginia Mason Medical Center, Seattle, WA 98101, USA; ^5^Endoscopy Unit, “A. Gemelli” University Hospital, Catholic University of the Sacred Heart, Largo A. Gemelli 8, 00168 Rome, Italy; ^6^Endoscopy Unit, Paoli-Calmettes Institute, 232 Boulevard de Sainte Marguerite, 13009 Marseille, France; ^7^Medicosurgical Department of Hepatogastroenterology, Saint-Philibert Hospital Centre, 59160 Lomme-lès-Lille, France; ^8^Gastroenterology, University of Alabama, Birmingham, AL 35233, USA; ^9^Gastroenterology, University of Pittsburgh Medical Center, Pittsburgh, PA 15213, USA

## Abstract

*Background*. Accurate diagnosis and clinical management of indeterminate biliary strictures are often a challenge. Tissue confirmation modalities during Endoscopic Retrograde Cholangiopancreatography (ERCP) suffer from low sensitivity and poor diagnostic accuracy. Probe-based confocal laser endomicroscopy (pCLE) has been shown to be sensitive for malignant strictures characterization (98%) but lacks specificity (67%) due to inflammatory conditions inducing false positives. *Methods*. Six pCLE experts validated the Paris Classification, designed for diagnosing inflammatory biliary strictures, using a set of 40 pCLE sequences obtained during the prospective registry (19 inflammatory, 6 benign, and 15 malignant). The 4 criteria used included (1) multiple thin white bands, (2) dark granular pattern with scales, (3) increased space between scales, and (4) thickened reticular structures. Interobserver agreement was further calculated on a separate set of 18 pCLE sequences. *Results*. Overall accuracy was 82.5% (*n* = 40 retrospectively diagnosed) versus 81% (*n* = 89 prospectively collected) for the registry, resulting in a sensitivity of 81.2% (versus 98% for the prospective study) and a specificity of 83.3% (versus 67% for the prospective study). The corresponding interobserver agreement for 18 pCLE clips was fair (*k* = 0.37). *Conclusion*. Specificity of pCLE using the Paris Classification for the characterization of indeterminate bile duct stricture was increased, without impacting the overall accuracy.

## 1. Introduction

The diagnosis of biliary strictures remains clinically challenging. Malignant strictures due to cholangiocarcinoma are particularly difficult to diagnose as they tend to grow slowly along the bile duct wall instead of forming mass lesions [1–3]. Biopsy, cytological brushing, and needle aspiration performed during Endoscopic Retrograde Cholangiopancreatography (ERCP) all suffer from low sensitivity and poor diagnostic accuracy [4–6]. Even when combining brush cytology with forceps biopsy, the sensitivity remains low at 54–71% [7–9]. Application of fluorescence in situ hybridization (FISH) in cytological brushing has offered increased hope in the investigation of indeterminate biliary stricture; however it requires dedicated laboratories with demonstrated experience for interpretation [10]. Direct visualization with cholangioscopy is helpful but is limited by the interobserver agreement [11, 12]. Lastly, positron emission tomography is not useful in early lesion without definitive mass [13].

As a result, there can be misdiagnosis or delays in confirming the diagnosis of biliary strictures. Delays in diagnosing malignant strictures place patients at risk for progression of disease precluding timely treatment [14].

Probe-based confocal laser endomicroscopy (pCLE) is an imaging technique used to provide in vivo histological information during endoscopy procedures. These microscopic images are obtained in real time by introducing a confocal miniprobe (CholangioFlex, Mauna Kea Technologies, France) through the working channel of a cholangioscope or a catheter [15]. The Miami Classification, an imaging criterion for pCLE diagnosis of malignancy in indeterminate strictures, has been shown to have an overall accuracy of 81% for diagnosis of malignant strictures compared to 75% for index pathology. However, due to false positives, the specificity of the Miami Classification is significantly lower at 67% [15].

**Figure 1:**
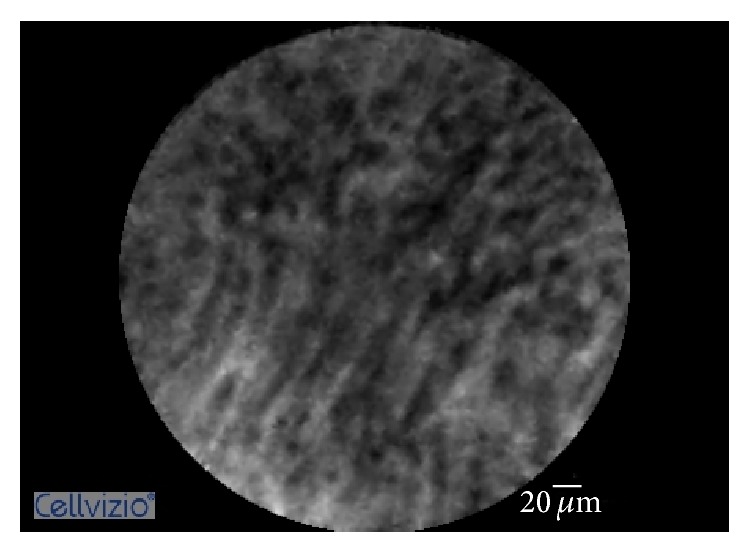
Probe-based confocal laser endomicroscopy imaging showing multiple thin white bands.

**Figure 2 fig2:**
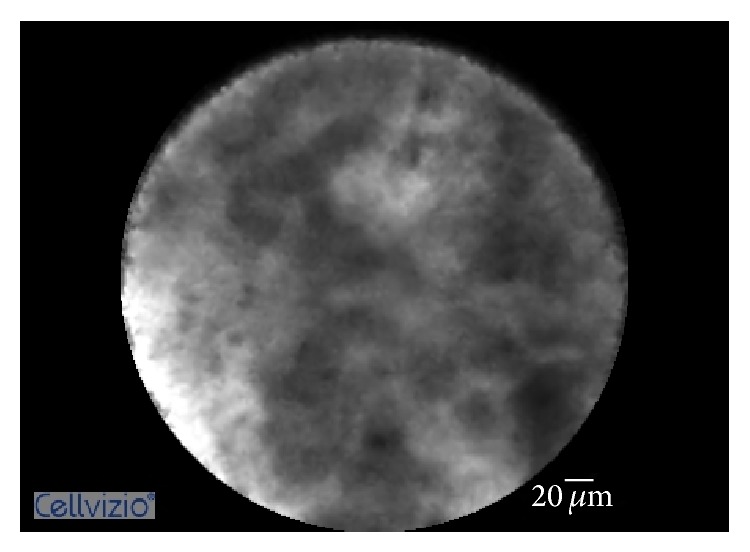
Probe-based confocal laser endomicroscopy imaging showing dark granular pattern with scales.

**Figure 3 fig3:**
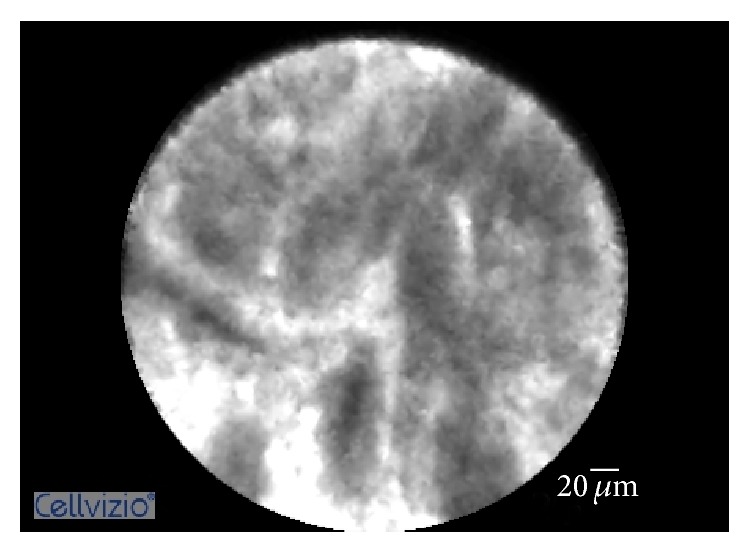
Probe-based confocal laser endomicroscopy imaging showing increased space between scales.

**Figure 4:**
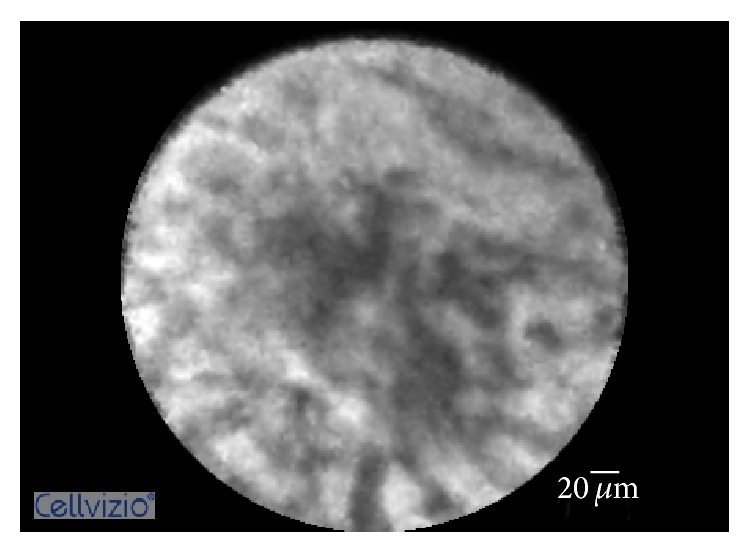
Probe-based confocal laser endomicroscopy imaging showing thickened reticular structures.

Until recently, no specific criteria existed to aid in the diagnosis of inflammatory strictures via pCLE. Caillol et al. recognized the limited ability of the Miami Classification to differentiate between malignant and inflammatory strictures and created a specific set of criteria, termed the Paris Classification [14]. The pCLE findings consistent with the diagnosis of inflammatory stricture are (1) multiple thin white bands, (2) dark granular pattern with scales, (3) increased space between scales, and (4) thickened reticular structures (Figures 1 [Fig fig2] [Fig fig3]–4). The objective of our study was to retrospectively evaluate the performance of pCLE for the diagnosis of inflammatory biliary stricture using the Paris Criteria.

## 2. Methods

Initially, 40 randomized pCLE videos/sequences with corresponding final diagnosis were acquired from a prospective registry containing 89 clips evaluating the diagnostic accuracy of pCLE for the characterization of bile duct stricture. These videos were reviewed by 3 pCLE experienced gastroenterologists who refined the existing Miami Classification by devising novel pCLE criteria for the characterization of inflammatory strictures, that is, Paris Classification. The 4 criteria devised for diagnosing inflammation in the bile duct were (1) multiple thin white bands, (2) dark granular pattern with scales, (3) increased space between scales, and (4) thickened reticular structures. These criteria were then reviewed and validated in consensus by 6 pCLE experts using a set of 40 pCLE sequences obtained during the prospective registry (19 inflammatory, 6 benign, and 15 malignant, based on final diagnosis). The corresponding interobserver agreement was further calculated on a set of 18 sequences (8 inflammatory, 9 malignant, and 3 benign) that had not been reviewed previously by any of the endoscopists rating those videos for interobserver agreement.

SAS 9.2 was used to conduct the statistical analyses. To measure agreement between study participants, we calculated Cohen's kappa coefficient before and after the teaching session. The results were interpreted based on the standards for strength of agreement proposed by Landis and Koch: poor agreement ≤0; slight agreement: 0 to 0.20; fair agreement: 0.21 to 0.40; moderate agreement: 0.41 to 0.60; substantial agreement: 0.61 to 0.80; almost perfect agreement: 0.81 to 1.0.

## 3. Results

Using the Paris Classification (PC), the overall accuracy in retrospectively diagnosing malignancy in 40 cases was 82.5% versus 81% (*n* = 89) for the prospective registry study, resulting in a sensitivity of 81.2% (versus 98% for the prospective study [16]) and a specificity of 83.3% (versus 67% for the prospective study [16]). The positive predictive value for the retrospective study was 76.5 versus 71% in the prospective study. The negative predictive value for the retrospective study was 86.9 versus 97% in the prospective study. The corresponding interobserver agreement (*n* = 6) for 18 pCLE clips was fair (*k* = 0.37).

Of note, when excluding one observer who had an accuracy of only 50% compared to 82.2% averaged by the remaining 5 observers, the interobserver agreement was good (*k* = 0.72).

## 4. Discussion

Diagnosis of indeterminate biliary strictures presents a clinical problem due to the low sensitivity of ERCP with tissue sampling. The low sensitivity is due in part to sampling the superficial epithelium [17]. Probe-based confocal laser endomicroscopy (pCLE) is an imaging modality that allows for targeted biopsy by offering in vivo histopathology and visualization of vasculature and architecture using fluorescein [17]. With the advent of the pCLE, the sensitivity of detecting biliary malignancies has increased from 50% with standard tissue sampling to 83% [17]. The increased sensitivity is counteracted by the decreased specificity (67% from 100%) given the increased false positives. The false positives can be attributed in large part to the misdiagnosis of inflammatory strictures caused by stenting, dilation, endoscopic procedure, or chronic inflammation as malignancies. Of note, the aforementioned statistics are with the use of the Miami Classification, an imaging criterion for pCLE diagnosis of malignancy in indeterminate biliary strictures. The criteria include loss of reticular pattern of epithelial bands of less than 20 *μ*m; detection of irregular epithelial lining, villi, or gland-like structures; tortuous, dilated, and saccular vessels with inconsistent branching; presence of “black areas” of more than 60 to 80 *μ*m (focally decreased uptake of fluorescein) [17, 18]. We sought to refine the Miami Classification to better differentiate between inflammatory strictures and malignancies and to reduce the number of false positives. The refined classification scheme is the Paris Classification and we attempted to validate it through a blinded retrospective review of pCLE videos.

Meining et al. in 2008 [16] in a 14-person study proposed that malignancy was indicated by loss of reticular pattern; irregular epithelium, villi, or glandlike structure; tortuous, dilated, and saccular vessels; and presence of black areas of more than 80 *μ*m indicating focally decreased uptake of fluorescein. By utilizing these criteria in conjunction with pCLE, it increased the sensitivity of detecting malignancy to 83% from 50% with traditional methods and had a specificity of 88%. Giovannini et al. in 2011 [18] in a 37-person study had similar criteria with malignancy being characterized as irregular vessels without contrast in the CBD wall, a large black band (>20 *μ*m), and irregular black cells/clumps and found the sensitivity and specificity for detecting malignancy to be 83% and 75%, respectively. Loeser et al. in 2011 [19] used the diagnostic hallmarks put forward by Meining in 2008 and found that the criteria were often nonspecific. In Meining et al. in 2012 [9], the Miami Classification was developed with malignancy being suggested by thick white bands (>20 *μ*m), thick dark bands (>40 *μ*m), or dark clumps or epithelial structures. The criteria were tested through blinded consensus review of 112 randomized pCLE videos from 47 patients and the sensitivity and specificity were found to be 97% and 33%. In Meining's 2011 multicenter study [15], the sensitivity and specificity of detecting cancer were 98% and 67%, respectively.

Given the low specificity ranging from 33% to 67%, the Miami Classification was refined and four descriptive criteria specific for benign inflammatory biliary features were stipulated [14]. The Paris Classification consists of the following: multiple thin white bands (vascular congestion), dark granular patterns with scales, increased spaces between scales (>20 microns), and thickened reticular structures.

In our study, by using the Paris Classification, the overall accuracy in retrospectively diagnosing malignancy in those 40 cases was 82.5% versus 81% (*n* = 89) for the prospective registry, the sensitivity was 81.2% (versus 98% for the prospective study), and the specificity was 83.3% (versus 67% for the prospective study). By using the Paris Classification in the retrospective study, we were able to improve the pCLE specificity by reducing the number of false positives. The specificity achieved was 83.3% compared to 67%. However, this came at a loss of sensitivity resulting in a lower negative predictive value. In our study, the interobserver agreement (*n* = 6) for 18 pCLE clips was fair (*k* = 0.37) but, with the exclusion of one observer with accuracy of only 50%, the interobserver agreement was good (*k* = 0.72). Thus, further physician training and interpretation standardization are required in order to improve interrater reliability of pCLE imaging.

Some of the limitations of the retrospective study include the following: the set of data reviewed by the investigators was limited to the information collected in the CRF of the prospective study and included the suggestive ERCP impression of the investigators. The pCLE video sequences were limited in time and arbitrarily chosen during the ERCP procedure, in real time, so as to reflect the presumptive diagnosis of the operator. However, it should be mentioned that histopathologists have access to the same information when reviewing tissue samples.

In conclusion, pCLE has the potential to overcome the limitations inherent to the ERCP procedure by providing real time, microscopic images of the bile duct epithelium, thus yielding a correct diagnosis more often than via ERCP. In vivo, real time histological imaging may open the door to immediate implementation of therapies [20].

Recognizing that benign inflammatory conditions can affect pCLE imaging and lead to misdiagnosis of malignancy and that the Miami Classification has a limited ability to differentiate between inflammatory conditions and malignancy, the Paris Classification was reevaluated and was found to have increased specificity of diagnosing indeterminate biliary strictures with pCLE without impacting the overall accuracy. There is a need for future studies, to evaluate each criterion of the Paris Classification scheme individually and set a threshold. In addition, the Paris Classification for pCLE should be prospectively tested in a larger number of sequences and is currently being evaluated in a prospective multicentric study.
